# Supplementary material for: The Th2 Response and Alternative Activation of Macrophages Triggered by Strongyloides venezuelensis Is Linked to Increased Morbidity and Mortality Due to Cryptococcosis in Mice
Source: J Fungi (Basel). 2023 Sep 26;9(10):968. doi: 10.3390/jof9100968 (PMC10607621; doi:10.3390/jof9100968)
Supplement: Supplementary file 1 [file jof-09-00968-s001.zip › jof-2564652-supplementary.pdf]

Supplementary Figure S1

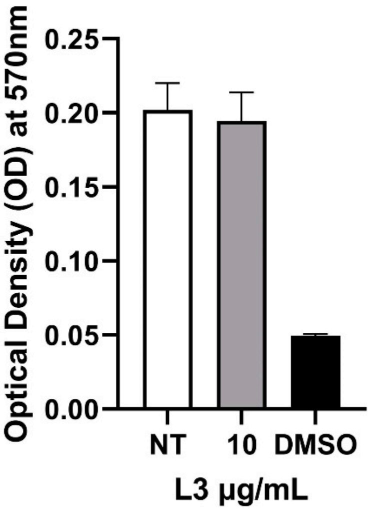

Figure S1: L3 antigen has low toxicity to murine macrophages. Viability of murine macrophages after exposure to L3 antigen. One-way ANOVA test/ Tukey's multiple comparison test. NT: control group, not exposed to L3; 10: macrophages exposed to 10 µ/mL of L3 antigen; DMSO: group exposed to dimethyl sulfoxide (DMSO). Data are representative of two independent experiments, and the results were always reproducible.
